# Videofluoroscopic measures of swallowing in people with stable COPD compared to healthy aging

**DOI:** 10.1590/2317-1782/20232022260

**Published:** 2023-10-23

**Authors:** Renata Mancopes, Catriona Margaret Steele

**Affiliations:** 1 Swallowing Rehabilitation Research Laboratory, KITE Research Institute - Toronto Rehabilitation Institute, University Health Network - Toronto (ON), Canada.; 2 Rehabilitation Sciences Institute, Temerty Faculty of Medicine, University of Toronto - Toronto (ON), Canada.

**Keywords:** COPD, Aging, Deglutition, Dysphagia, Videofluoroscopy, Kinematics

## Abstract

**Purpose:**

Swallowing impairment is a serious extra-pulmonary manifestation of Chronic Obstructive Pulmonary Disease (COPD). Previous studies suggest that individuals with stable COPD show atypical values for several videofluoroscopy measures of swallowing, compared to healthy adults under age 60. However, it is unclear to what degree these changes are attributable to healthy aging. In this study, we aimed to clarify how swallowing in people with stable COPD differs from age-matched healthy controls.

**Methods:**

We performed a retrospective analysis of videofluoroscopy data from two previously-collected datasets: a) a sample of 28 adults with stable COPD (18 male); b) a sample of 76 healthy adults, from which 28 adults were selected, matched for sex and age to participants in the COPD cohort. In both prior studies, participants swallowed 20% w/v liquid barium prepared in different consistencies (thin; mildly, moderately, and extremely thick). Blinded duplicate ratings were performed according to a standard procedure, yielding measures of laryngeal vestibule closure (LVC) integrity and timing, swallow timing, upper esophageal sphincter (UES) opening, pharyngeal constriction and pharyngeal residue. Mann-Whitney U tests and odds ratios were performed to determine significant group differences (p<.05).

**Results:**

Across the consistencies tested, participants with COPD showed significantly shorter durations of LVC, earlier onsets and shorter durations of UES opening, and reduced pharyngeal constriction. No significant differences were seen in other measures.

**Conclusion:**

These results point to features of swallowing in people with stable COPD that differ from changes seen with healthy aging, and which represent risks for potential aspiration.

## INTRODUCTION

Chronic Obstructive Pulmonary Disease (COPD) is a serious public health problem involving persistent respiratory symptoms and airflow limitation that are caused by airway abnormalities, typically arising from exposure to noxious particles or gases^(1)^. Prior to the COVID-19 pandemic, COPD was projected to be the 3^rd^ leading cause of death internationally by 2021^(1)^. Several studies suggest that dysphagia (swallowing impairment) may be an extrapulmonary feature of COPD^(2-8)^. However, there is a need to clarify whether and how changes seen in swallowing in COPD differ from those seen in healthy aging^(9,10)^. Individuals with COPD are thought to be prone to dysphagia as a result of altered breathing patterns and abdomino-thoracic biomechanics, especially during phases of exacerbation^(11-13)^.

In a recent study, Mancopes et al.^(9)^ performed a detailed analysis of videofluoroscopic measures of swallowing safety, efficiency, timing and kinematics in a sample of 28 adults with stable COPD, compared to reference data for healthy adults aged under 60. Although increased rates of airway invasion (“penetration-aspiration”) were not observed in the COPD cohort, the authors identified significantly higher frequencies of atypical scores for parameters related to airway protection, including incomplete closure of the entrance to the airway, henceforth referred to as “laryngeal vestibule closure” (LVC), prolonged time-to-LVC and short LVC duration. Additionally, an increased prevalence of poor bolus clearance in the form of pharyngeal residue was observed in the COPD cohort, and this was particularly associated with the combination of poor pharyngeal constriction and shortened duration of upper esophageal sphincter (UES) opening.

It remains unclear whether any of these changes may be explained by healthy aging (“presbyphagia”). Mancopes et al.^(10)^ have recently published a second analysis of age-related changes in videofluoroscopic measures of thin liquid swallowing in a cohort of 76 healthy adults, aged 19-82. Hierarchical linear regression models showed no effect of age on time-to-LVC, the timing of UES opening relative to swallow initiation (“hyoid burst onset”), or measures of pharyngeal residue. However, older age was significantly associated with prolonged swallow reaction time (i.e., time to swallow initiation), and worse pharyngeal constriction. Additionally, healthy older adults were noted to display three changes suggesting possible spontaneous compensation for slower bolus transit: prolonged durations of LVC, prolonged durations of UES opening, and wider UES opening diameter.

The objective of this paper is to clarify whether there are changes in swallowing in stable COPD that are distinct from those seen in healthy aging. To answer this question, we used data from the original Mancopes et al. study in individuals with stable COPD^(9)^ and data from the Mancopes et al.^(10)^ study of healthy aging. We matched each participant in the COPD cohort to healthy age- and sex-matched control and compared measures of swallowing physiology across the full range of liquid bolus consistencies from thin to extremely thick liquids. We hypothesized that we would see differences in measures of airway protection in those with COPD, including higher frequencies of incomplete LVC and shorter durations of LVC, and we also expected to see shorter durations of UES opening in the COPD cohort. Furthermore, given that muscle weakness is known to occur in COPD^(14)^, we hypothesized that these individuals would display significantly reduced pharyngeal constriction compared to the healthy controls.

## METHODS

This study involved a retrospective of two previously collected datasets^(9,10)^. Data for individuals with stable COPD were available for 28 adults (18 male) who were patients of the pulmonary rehabilitation program at the University Hospital of Santa Maria (Ethical approval number 1.967.549). Data for 28 healthy age-and sex-matched controls were extracted from a healthy reference dataset collected at the University Health Network (Reb Approval number15-9431-D). Participants in both prior studies provided written informed consent, and the study protocols were approved by the local institutional ethics review boards.

Videofluoroscopy data were acquired at 30 pulses per second and recorded at 30 frames per second. Participants swallowed 20% w/v concentration barium in thin, mildly, moderately, and extremely thick consistencies, prepared with a xanthan gum thickener (Resource Thicken Up Clear, Nestlé Health Science), as described in the original manuscripts^(9,10)^. Participants took comfortable sips and swallowed without waiting for a cue from the investigator. To match the number of boluses for comparison across datasets, measures for the first bolus of each consistency were used.

As described in the original manuscripts, the videofluoroscopy recordings were rated in duplicate by trained raters, blinded to consistency and each other’s ratings, according to a standard procedure known as the ASPEKT Method^(15)^. Discrepancies were resolved by consensus. This process yielded measures of airway protection (penetration-aspiration, LVC integrity and timing), swallow timing, UES opening diameter, pharyngeal constriction and pharyngeal residue. The parameters of interest for this study are listed in Table 1.

**Table 1:** Parameters measured in this study according to the ASPEKT Method^(15)^

**Parameter**	**Unit of measure**	**Definition**
Laryngeal Vestibule Closure (LVC) Integrity	Categorical	Degree of laryngeal vestibule closure: complete/incomplete
Time to LVC	ms	Interval from hyoid burst onset (HYB) until LVC.
LVC Duration	ms	Interval from LVC until opening of the laryngeal vestibule.
Swallow Reaction Time	ms	Time from bolus passing mandible to HYB
Hyoid burst onset to upper esophageal sphincter (UES) opening	ms	Interval from HYB until upper esophageal sphincter opening
UES opening duration	ms	Interval from onset of UES opening until UES closure
Pharyngeal area at maximum constriction	%(C2-4)^2^	Pixel-based measure of the 2-dimensional lateral area of the pharynx on the frame of maximum pharyngeal constriction, normalized to the squared length of a cervical spine scalar (distance between the anterior inferior corners of C2 and C4)
Total pharyngeal residue	%(C2-4)^2^	Sum of residue area measures for the valleculae, pyriform sinuses and elsewhere in the pharynx

Difference scores between paired COPD participants and healthy controls were computed and inspected for normality. In almost all cases, the distributions were skewed. Therefore, we performed nonparametric Mann-Whitney U Tests to determine whether there were significant differences between groups for continuous parameters, by consistency. LVC Integrity was the only categorical parameter, and for this, we used cross-tabulation, Fisher’s exact tests and odds ratios to examine differences in the frequency of incomplete LVC. All statistical analyses were performed in SPSS version 28 software, using a p-value of 0.05.

## RESULTS

**Figure 1:**
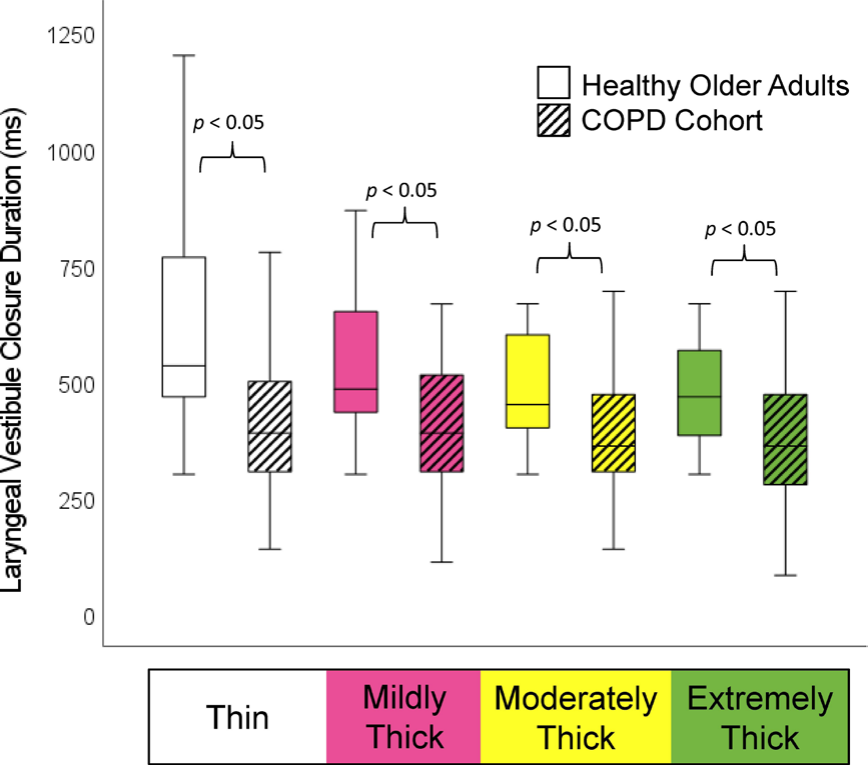
LVC Duration by Consistency and Cohort. Participants with COPD displayed significantly shorter laryngeal vestibule closure (LVC) durations on all consistencies

**Figure 2:**
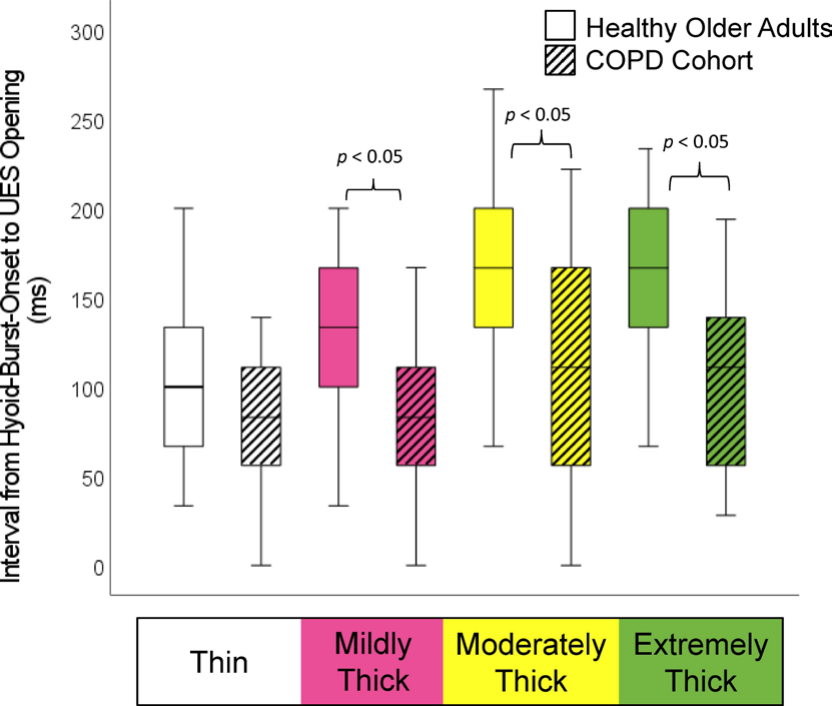
Hyoid-Burst-Onset to UES Opening Interval by Consistency and Cohort. Participants with COPD displayed significantly earlier upper esophageal sphincter (UES) opening relative to onset of the hyoid burst on mildly, moderately and extremely thick liquids

**Figure 3:**
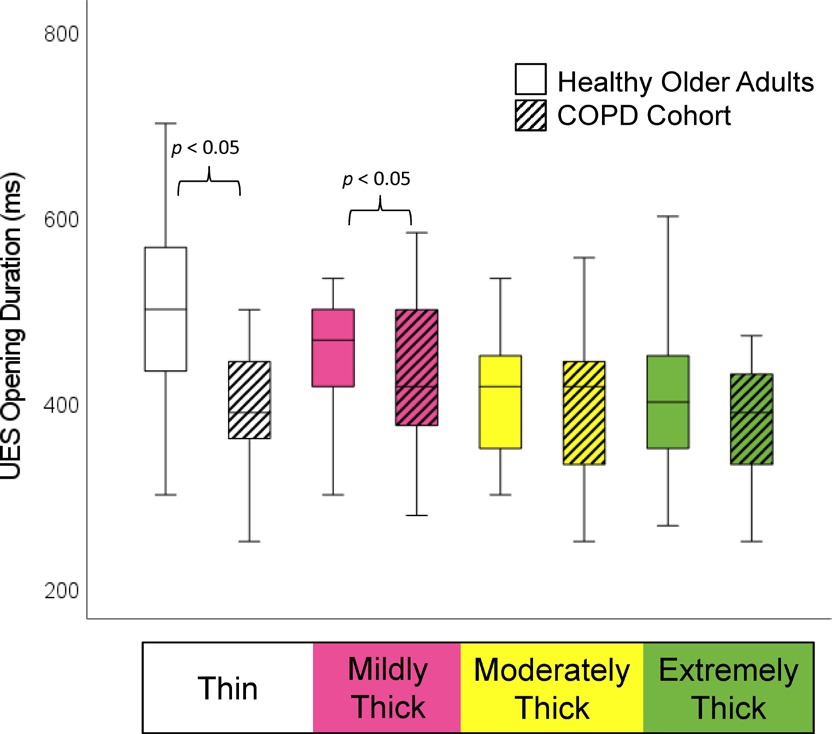
UES Opening Duration by Consistency and Cohort. Participants with COPD displayed significantly earlier upper esophageal sphincter (UES) closure relative to onset of the hyoid burst, and significantly shorter UES opening durations on thin and mildly thick liquids

**Figure 4:**
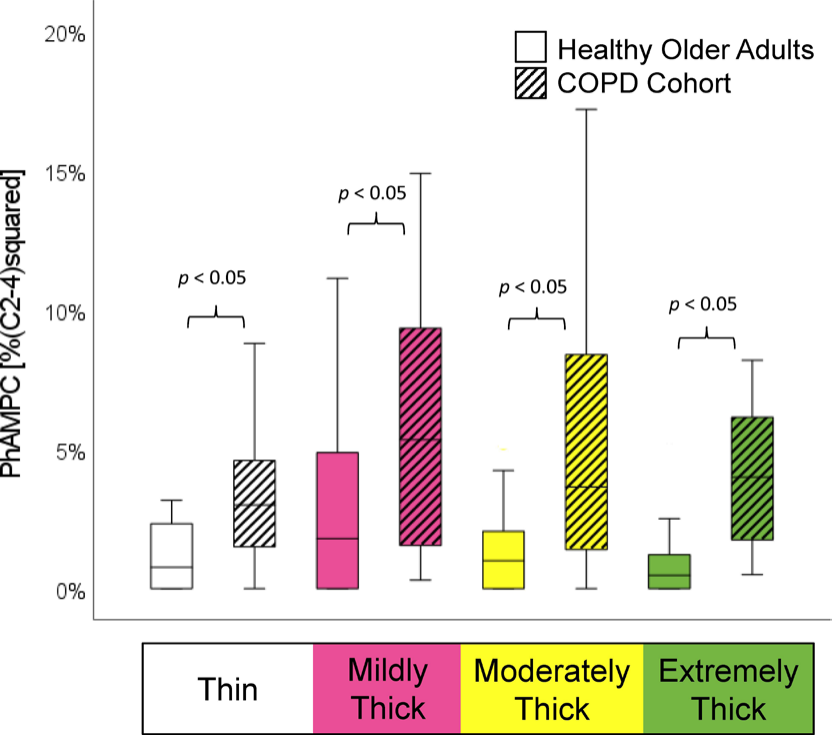
Pharyngeal Area at Maximum Constriction by Consistency and Cohort. Participants with COPD displayed significantly poorer pharyngeal constriction, as reflected by larger pharyngeal area at maximum constriction, on all consistencies. Pharyngeal area was measured in anatomically scaled units, normalized to the squared length of the C2-C4 spine

The demographic and respiratory characteristics of the study participants are presented in Table 2. As reported in the original manuscript, the COPD patients did not present with increased frequencies of penetration, and none of them aspirated material below the level of the vocal folds. As shown in Table 3, incomplete LVC was more common in the COPD cohort, but this failed to achieve statistical significance. Significant differences were found in the form of shorter LVC duration, shorter hyoid-burst-to-UES-opening duration, and shorter UES opening duration in the COPD cohort, along with reduced pharyngeal constriction (i.e., larger pharyngeal area at maximum constriction). These differences are shown by consistency in Figures 1-4.

**Table 2:** Participant Demographics.

**Parameter**	**COPD (n=28)**	**Healthy Controls (n=28)**
Mean Age	65	65
Age Range	41-79	48-82
FEV_1_% predicted (Mean ±SD)	44±18	N/A
FVC % predicted (Mean ±SD)	70±22	N/A
FEV_1_/FVC	48±12	N/A
GOLD 1 (%, n)	3.6% n=1	N/A
GOLD 2 (%, n)	25% n=7	N/A
GOLD 3 (%, n)	53.6% n=15	N/A
GOLD 4 (%, n)	17.9% n= 5	N/A
Body Mass Index (Mean ±SD in kg/m^2^)	25.5±5.1	N/A

Caption: FEV_1_ = Forced Expiratory Volume in the 1st second; FVC = Forced Vital Capacity; GOLD = Global Initiative for Chronic Lung Disease Severity Staging (2021); N/A = Not measured

**Table 3:** Frequency of Incomplete Laryngeal Vestibule Closure (LVC) by Consistency and Cohort

**Bolus Consistency**	**Cohort**	**Incomplete LVC**	
		**N**	**% within cohort**
Thin	COPD	7	25%
	Controls	2	7%
Mildly thick	COPD	6	21%
	Controls	1	4%
Moderately thick	COPD	4	15%
	Controls	0	0%
Extremely thick	COPD	1	4%
	Controls	0	0%

## DISCUSSION

Dysphagia is an under-recognized comorbidity in COPD, and the pathophysiology of swallowing impairment in COPD is not yet well established^(16)^. The purpose of this study was to clarify the nature of changes in swallowing in stable COPD as compared to age-matched healthy controls. The majority of participants in the COPD cohort were classified as having disease severity at GOLD Level 3, which corresponds to severe airflow limitation with forced expiratory volume measures between 30% and 50% of predicted values^(1)^. However, it is important to emphasize that these participants were in a stable disease phase rather than experiencing exacerbation. The absence of penetration-aspiration seen in our COPD cohort represents a lower frequency of airway invasion than that described in prior studies by Garand et al.^(5)^, Cvejic et al.^(17-20)^ and others^(3,21-23)^, but is consistent with other studies in which penetration-aspiration has not been seen in patients with stable COPD^(2,4,6,24)^. Here, both demographic and methodological differences across studies appear likely to contribute to the differences in results. In one recent study, Garand et al.^(5)^ enrolled a cohort of 10 adults aged 53-76, described to have stable but advanced-stage COPD but also to be underweight, with body mass index (BMI) below 22 kg/m^2^. By comparison, only 8 of the 28 participants in our COPD cohort had BMI values below that cut-off. Garand et al.^(5)^ also reported that none of the participants in their COPD cohort showed penetration-aspiration on discrete single sips of liquid barium (from thin to extremely thick consistency). Rather, episodes of penetration (n = 2) and aspiration (n = 1) were isolated to sequential drinking tasks of thin and nectar-thick consistency. This concurs with evidence from the series of recent studies by Cvejic et al.^(17-20)^, who reported a 20% frequency of aspiration in 151 adults with stable COPD when drinking 100 ml volumes of thin liquid, either as a series of self-paced discrete sips or by continuous drinking. Thus, the frequencies of penetration-aspiration observed on the initial discrete sips of each consistency in our study may under-represent the true frequency of airway invasion in stable COPD. Notably, the work by Cvejic et al. ^(18,19)^ identifies an association between aspiration observed during their 100 ml thin liquid swallowing challenges and the frequency of acute COPD exacerbations.

Notwithstanding the fact that penetration-aspiration was not observed in the participants with COPD, our analysis did show significantly shorter durations of LVC across all consistencies in the COPD cohort, which may represent an increased risk for airway invasion. These same participants were previously reported to also show an increased frequency of incomplete LVC and prolonged time-to-LVC compared to reference values for healthy younger adults. In the current analysis, the frequency of incomplete LVC was greater in the COPD cohort than in age-matched controls, but this difference was not statistically significant. Additionally, the non-parametric comparison of time-to-LVC did not show significant differences.

Previous studies have reported observations of prolonged pharyngeal transit time in patients with COPD^(2,4)^. The studies by Cvejic et al.^(18)^ also describe prolonged overall time to complete the 100 ml liquid swallowing challenge in patients with aspiration, regardless of the task instructions (discrete vs. rapid sequential drinking). In the prior comparison of data from our COPD cohort to reference values for healthy younger adults, Mancopes et al.^(9)^ reported significantly increased frequencies of prolonged swallow reaction time and pharyngeal transit time on thin liquids in the patients with COPD. In the current non-parametric comparison of values to age-matched controls, we no longer found any significant differences in swallow reaction time for any consistency. When other timing measures were compared, relative to the frame of hyoid burst onset, the COPD cohort did not show longer latencies than the age-matched controls, but rather showed the opposite pattern of significantly earlier UES opening (mildly, moderately and extremely thick consistencies), and significantly earlier UES closure (all consistencies), as illustrated in Figures 2 and 3. This latter finding corresponds with significantly shorter UES opening durations, a finding that was also seen in the previous comparison with healthy younger adults. ^9^The reasons for these discrepancies in findings compared to prior literature are not clear, but may reflect differences in study methodology, bolus volumes and measurement definitions.

As hypothesized, our analysis showed significantly poorer pharyngeal constriction in the COPD cohort compared to age-matched controls, across all consistencies. Poor pharyngeal constriction in COPD was noted in our earlier investigation as a dramatic but previously unreported characteristic of swallowing in COPD^(2,4,5)^. In the earlier comparison to healthy younger reference values, the frequency of atypical residue (i.e., above the 3^rd^ quartile reference value) was found to be significantly increased in the COPD cohort on the mildly and extremely thick consistencies. Poor pharyngeal constriction in COPD may be related to muscle weakness that is characteristic of the disease and thought to arise from the effects of systematic inflammation, smoking, hypoxemia, chronic inactivity, oxidative stress and malnutrition^(25-27)^. Additionally, force generation during swallowing may be reduced as a result of hypercontraction and shortening of diaphragm muscle fibers^(28)^. A recent study by Sugiya et al. ^(14)^ further suggests that there may be an association between tongue weakness, swallowing impairment and whole body skeletal muscle mass change in COPD. Given that muscle dysfunction is a major complication for COPD patients, future studies should explore these relationships in more detail.

As with any study, this study is not without limitations. As noted already, the data collection protocol was limited to discrete, comfortably sized natural sips and did not challenge the system with a large volume or sequential drinking task. The data are also limited to videofluoroscopic parameters, and did not include monitoring of respiration or respiratory-swallow coordination. Abnormal timing of swallows within the respiratory cycle with an increased frequency of swallows followed by inspiration has been identified as a pattern that may be seen more commonly in people with COPD and linked to penetration-aspiration and disease exacerbation^(7,29,30)^. In addition, the number of participants was limited and insufficient to permit generalization.

## CONCLUSION

This retrospective analysis compared videofluoroscopic measures from discrete swallows of liquids across the range from thin to extremely thick consistency in patients with stable COPD and age-matched healthy controls. Data from the COPD cohort showed significant differences in the form of shortened laryngeal vestibule closure, earlier and shorter upper esophageal sphincter opening, and markedly reduced pharyngeal constriction. Although penetration-aspiration and pharyngeal residue were not observed in this particular COPD cohort, the findings point to features of swallowing in people with stable COPD that differ from changes seen with healthy aging, and which represent risks for potential aspiration.
